# Combining market surveys and participative approaches to map small ruminant mobility in three selected states in northern Nigeria

**DOI:** 10.1371/journal.pone.0311030

**Published:** 2025-09-02

**Authors:** Sandra I. Ijoma, Asma Mesdour, Muhammad-Bashir Bolajoko, Chika Nwosuh, Marion Bordier, Arnaud Bataille, Adeiza M. Abdulrahman, Wesley D. Nafarnda, Elena Arsevska, Andrea Apolloni

**Affiliations:** 1 Veterinary Public health and Preventive Medicine Department, National Veterinary Research Institute, Vom, Plateau state, Nigeria; 2 Joint Research Unit Animals, Health, Territories, Risks, and Ecosystems (UMR ASTRE), University of Montpellier, French Agricultural Research Centre for International Development (CIRAD), National Research Institute for Agriculture, Food and Environment (INRAE), Montpellier, France; 3 Joint Research National Laboratory for Livestock and Veterinary Research, Senegalese Institute of Research in Agriculture, Dakar, Senegal; 4 Joint Research Unit Animals, Health, Territories, Risks, and Ecosystems (UMR ASTRE), French Agricultural Research Centre for International Development (CIRAD), Montpellier, France; 5 University of Abuja, Federal Capital Territory, Abuja, Nigeria; University of Uyo, NIGERIA

## Abstract

In Nigeria, a huge gap in knowledge on livestock mobility and its role on transboundary disease spread exists. As animals move, so do diseases. Therefore, there is a need to understand how livestock movements can contribute to the circulation and maintenance of infectious livestock diseases which can impede the design of particular surveillance and control tactics in the event of outbreaks. Our study aim was to reconstruct small ruminants’ mobility patterns in three selected states in Northern Nigeria for better surveillance and control of small ruminant’s transboundary animal diseases (TADs). To this end, a mixed approach was used to collect data. A market survey, employing structured questionnaires, was administered to 1,065 market traders. Additionally, 20 focus group discussions were conducted with traders and transhumance actors across 10 Local Government Areas (LGAs) spanning three northern Nigerian states: Plateau, Bauchi, and Kano. The respondent movements by type, animal movement, reason for movement was described and summarized. Data collected were used to reconstruct small ruminant mobility networks, whose nodes were LGAs, in the three states of the survey area and with other states in Nigeria and movement mapped. Characteristics of both networks were studied using a complex network approach either separately or combined. Using the two approaches provided a complementary view of small ruminant mobility. The reconstructed networks were connected, highly heterogeneous and had very low density. The networks included LGAs belonging up to 31 states. The presence of hubs increased the risk of disease spread. Gwarzo, Wudil (Kano) and Alkaleri (Bauchi) LGAs received the most sheep and goats, while Jos North (Plateau) and Gwarzo supplied more small ruminants. Bukuru and Alkaleri markets were classified as super-spreaders with a higher probability of detecting virus circulation. Four to six multistate communities were identified. Our findings could support policy choices to identify priority areas for surveillance and disease control in small ruminants.

## Introduction

Livestock mobility in Sub-Saharan Africa is considered as one of the most significant factors contributing to the spread of several transboundary animal diseases (TADs) [1,2]. In a very broad way, two types of live animal movements exist in Sub-Saharan Africa: transhumance and commercial. Transhumance movements are seasonal migrations driven by the search for grazing land, while commercial movements involve the live sale or trade of livestock to meet household needs, red meat demand, and cultural and religious obligations [2]. In Nigeria, cross-border trade of livestock has been occurring through formal and informal routes from Benin, Cameroon, Chad, and Niger, to supply markets in Nigeria in exchange for other goods from Nigeria [3,4]. At the same time Nigerian farmers, herders and traders often access markets situated in the same area or in other LGAs (representing the administrative Unit of Level 2 in Nigeria), or even in another state (representing the administrative Unit of Level 1) to exchange animals and product [4].

Animal mobility is driven by a complex interplay of ecological, socio-economic, and cultural factors [1,5–8]. From the ecological point of view, movement is essential for exploiting grazing resources to avoid overgrazing [9]. It also serves as a climate change adaptation strategy: due to severe environmental conditions, farmers counter the variability of pasture and water across the year by migrating with their herd during the beginning of the dry season from the Northern region to the southern region and return at the beginning of the rainy season [10,11]. From the socio-economical point of view, live intra-regional animal trade dominates the region of West Africa despite the high costs for transporting and handling animals, sustained by policies that impede processing [12]. Rural affordability and accessibility favor live animal trade, whereas urban processed meat growth is limited by traditional methods, price, supply chain complexity, and infrastructure deficits [12–15]. Additionally, live animals trade and mobility represent a choice of livestock owners to mitigate disease events [16–19] by destocking herds or sending them away to protect from infection. These factors are particularly relevant in Nigeria, where consumers often prefer to select live animals for freshness and quality, and where cultural and religious traditions often involve home slaughter [15].

Nigeria has one of the largest small ruminant populations in Africa, with over 76.3 and 48.6 million goats and sheep, respectively in 2022 [20,21]. Over 70% of the small ruminant population is found in the Northern region, with over 8 and 3 million, respectively, in Bauchi and Plateau [20,22,23]. Small ruminants are typically reared in either extensive transhumant (pastoral), or sedentary (backyard) systems [20,24]. Small ruminants contribute to people’s livelihoods within Nigeria through employment, wealth generation as most rural households and farmers use them as banks [20].

The persistent occurrence of diseases TADs in rural communities such as Peste des Petits Ruminants (PPR), Foot and Mouth disease (FMD), Sheep and Goat pox (SGP), Lumpy Skin disease (LSD) [1,25] has threatened economic and food security, thereby jeopardizing their livelihood [24,26]. Among small ruminant diseases of importance to Nigeria is PPR, which has been present in the country since at least 1975 [20,27–36]. PPR is targeted for eradication by 2030 [26]. Within the framework of the global eradication program coordinated by the Food and Agriculture Organization of the United Nations (FAO) and the World Organization for Animal Health (WOAH) [26] that aim to control by reinforcing surveillance system and vaccination campaigns [28,36]. Nigeria produces PPR vaccines, but control efforts are still limited and continuous outbreaks of PPR are a cause of concern [36]. Better knowledge of livestock mobility patterns could help identify areas/markets to monitor for surveillance and control of introduction and spread of PPR and other TADs.

Although some research exists on commercial livestock mobility in cattle, pigs, and poultry, a significant knowledge gap remains regarding small ruminant mobility in both Nigeria and other regions [3,37–42]. This lack of understanding is further compounded by porous borders and the absence of a centralized livestock identification and tracking system, hindering effective monitoring and control of transboundary animal diseases (TADs). Nevertheless, these factors hinder the possibility of relying on quantitative data collected routinely and new mixed approaches need to be put in place to collect and analyze data.

Globally, studies employed network analysis to characterize animal mobility patterns and assess disease spread risks associated with livestock trade networks [43–45]. This helps identify areas and populations most vulnerable to disease outbreaks [37,38]. The 2001 UK FMD outbreak was one of the first instances where network analysis was used retrospectively to analyze disease spread patterns [43].

Recently, some studies have used network approaches in West Africa [1,5,25,40,46–48] with the aim of mapping and analyzing livestock movements in the area. Nevertheless, this analysis requires data at market through ad hoc activities and provides a partial view of the mobility network. To fill the gaps, participative approaches [49–52] could present a viable means to collect data and provide a description of the general patterns in a specific area.

Therefore, the objective of this study was to describe and map small ruminant movement in selected northern Nigerian states by integrating market survey and participatory approach to identify market linkages, hotspots, and network structures for better surveillance and control of small ruminant’s infectious diseases, with particular interest in PPR, FMD and Sheep and Goat Pox. This study’s findings can inform targeted surveillance and control strategies for small ruminant TADs, particularly PPR, FMD and Sheep and Goat Pox, which continues to pose a significant threat to livelihoods and national development.

## Materials and methods

### Study area

**Fig 1:**
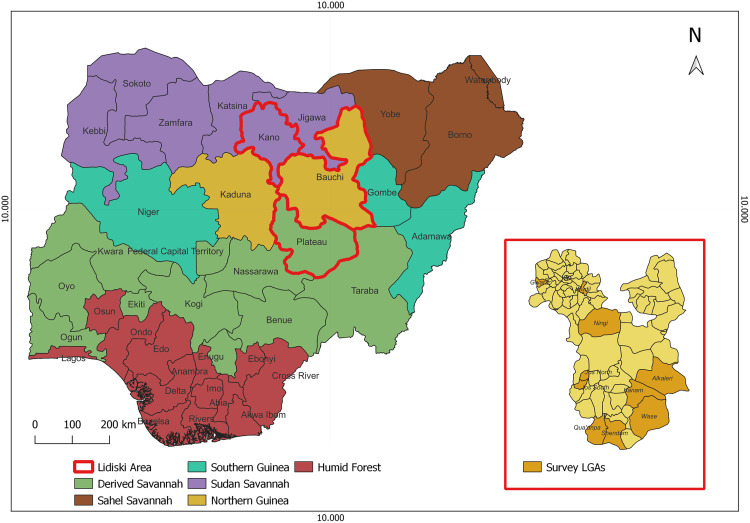
Agro-ecological zones of Nigeria and the three study states of the Lidiski project (Plateau, Bauchi and Kano), and their corresponding LGAs (inset).

Nigeria is a West African country located between 4- and 14-degree North latitude on the equator and between longitude 3- and 15-degree East longitude delimited on the Greenwich Meridian and is bounded on the South by the Gulf of Guinea, on the East by Cameroon, on the North by Chad and Niger and the West by Benin. The great extent of the territory justifies the presence of six agro-ecological zones. The Northern part of the country is characterized by a Sahelian type of climate with low rainfall duration, and longer dry seasons [53]. The Southern is humid and tropical with some areas characterized by a tropical monsoon climate with heavy rain (March-October) and high humidity, and others a tropical savanna climate with a single rainy season (April-September) and hot, dry season influenced by the Harmattan wind [53]. The differences in climatic condition and rainfall are main factors for transhumant movements [53]. From the administrative point of view, Nigeria is a federal country divided into 36 states and the Federal Capital Territory, which are further divided into LGAs subdivided into districts/wards (Fig 1). The majority of the small ruminants’ population is concentrated in the states in the Northern arid areas and in the South East [18,20,54].

The study was conducted under the framework of the Lidiski project [55], whose main objective is to improve surveillance and control of PPR affecting the livestock of smallholder farmers in Nigeria [55] and whose intervention area includes three states in the Northern Area (Plateau, Bauchi and Kano). These states were selected based on previous research that showed PPR existed there, the highest prevalence rate of PPR in this region [31,36,56,57], the high presence of sheep and goat population and concentration of livestock movements in the area that could contribute to the introduction and spread of TADs [10,22]. These survey states correspond to the three out of the six agro-ecological zones in Nigeria [36], i.e., Northern Guinea, Sudan Savannah, and Derived Savannah agro-ecological zones, respectively (Fig 1). In this area, a large proportion of the population is constituted by subsistence farmers consisting of either pastoralists or agro-pastoralists who keep small ruminants for household consumption and commercial purposes [24]. Nevertheless, the three states are situated along a few main axes of transhumant and commercial movements from the northern border with Nigeria to Abuja and the Southern regions, and from the eastern border with Cameroon towards the central area [11].

### Data collection

#### Selection of surveyed markets.

A focus group discussion meeting (FGD) of stakeholders was held at the National Veterinary Research Institute, Vom, Nigeria in April 2021, to identify main ruminant markets in Plateau state. Using purposive sampling [58], fifteen stakeholders who were very knowledgeable about livestock markets including, researchers, veterinary services officers, representatives of market leaders and transhumant herders, were gathered and asked to identify main ruminant markets in Plateau state. In a second step they were asked to draw the connections among the animal movements between markets, thus providing the skeleton of the mobility patterns. Based on the preliminary analysis of these network movements, the centrality of the market in the trade network, the markets with the highest supply of small ruminants, and on logistical and security constraints, we selected a subset of six major small ruminant markets to be surveyed. Following the same approach as for Plateau and based on their connection to Plateau state, two major small ruminant markets in Kano and in Bauchi were then selected. Altogether, a total of 10 markets located in 10 different LGAS among the three states were selected to be surveyed. The market surveys and FGDs were conducted from April 1, 2022 to September 30, 2022. Information on the survey objectives and consent forms were read with the participants. An oral informed consent was obtained from all participants in the surveys and the FGDs. All personal data were anonymized at the time of collection. In each LGA, market mobility data were collected using two complementary approaches: market survey (individual interviews with respondents) and focus group discussions. For the market survey and FGDs, participants were traders who sell or buy goat and sheep or have some involvement in small ruminant trade such as transporters, market leaders, the owners of animals, or the ones responsible for the sale of animals or products while those who were not involved in small ruminant trade were excluded. Before the surveys and FGD took place, an advocacy activity was carried out with market leaders and transhumant leaders so that they could be involved in the activity and purposefully identify respondents and participants to the FGD. Market survey respondents identified themselves in one of the categories: Traders as people who sold or bought sheep and goats for the purpose of reselling the small ruminants again; Middle men were those in the market who connected the sellers with the buyers of sheep and goats; Transporters were those who moved the sheep and goats from one market to the other on the behalf of the traders; Active transhumance or pastoralists were defined as nomadic herders who migrate with their small ruminants seasonally every year.

#### Market survey (Individual interviews).

A total of 1,065 individuals (among traders, transporters, farmers) were successfully interviewed across the 10 LGA from April 1, 2022 to September 30, 2022. A convenience sampling approach was employed, wherein participants were approached for voluntary participation during their presence at the market. Respondents were asked to provide information about the most precise place of origin and destination of the animal movement (Country, state, LGA, village), species transported (goats, sheep or both), number of animals transported, frequency of market visits and reason for movement. Individual, questionnaire-based interviews were conducted with structured questionnaires at participants’ convenience to maximize response rate.

#### Focus group discussions.

To address the convenience sampling selection bias from the market survey, obtain a more comprehensive picture of mobility patterns and get insight about other possible driving factors of mobility, a second round of FGDs were organized in April 1, 2022 to September 30, 2022 with the transhumance groups and the small ruminant trader groups in each of the same 10 LGAs where a market was surveyed. FGDs were organized separately for market actors (small ruminant traders and transporters) and transhumant herders. In each FGD, 10 participants attended for a total of 200 participants. Using open- ended questions and participatory tools (proportional piling, seasonal calendars, and maps), we collected participant knowledge about animal origins and destinations, the period of the year when movements were occurring and the reason behind the choice of specific markets. Each interview lasted up to 2 hours and involved a team of three researchers (facilitator, note-takers). In 2022, FGDs meetings with the transhumance groups and the small ruminant trader groups were held in these same 10 LGAs and three selected states to complement the information [59] on animal movements. For the market survey and FGDs, participants were traders who sell or buy goat and sheep or have some involvement in small ruminant trade such as transporters, market leaders, the owners of animals, or the ones responsible for the sale of animals or products while those who were not involved in small ruminant trade were excluded.

### Data management

#### Market survey.

Data were collected using an electronic-based questionnaire developed on the Kobo collect platform. FGD data were collected on flipcharts and then transferred to spreadsheets. For mobility data (both collected with FGD and market survey), they were recorded as edge-list, where each line corresponds to a movement between an origin and a destination, for each species as reported by the respondent. Data of the place of origin and place of destination (state, LGAs and villages) were cleaned, spellings were standardized and locations were verified using Wikipedia and Google searches. Then using queries on Google maps and search on the internet, LGAs were geo-localized and coordinates associated to each village recorded. The LGA was selected as the basic administrative unit for this survey.

#### Statistical analysis.

Exploratory analysis was done for both FGD and market survey data to provide an overview of the data collected, the characteristics of the respondents, the extension of the areas involved in the movements, the frequency of the movements and the total number of animals exchanged. The type of movements, reason for both actor and animal movements and the direction of animal movements were also described and analyzed. Fisher test was used to find if there was a significant relationship between the movements of actors and their type of movements inside and outside the survey areas.

#### Network analysis.

Mobility data were aggregated at LGA level, which represented the nodes of the network, and a link represented a movement between two LGAs. Three networks were created: a direct weighted network from the market survey, whose links’ weight corresponds to the quantity of animals traded with the given link (market network); a direct unweighted network reconstructed from the FGD (FGD network); a direct network formed by the union of the FGD and the market ones, without taking in account the weights of the links (combined network).

Analyses were performed for each network separately and then for the combined one. Jaccard index [25] measures for links and nodes present in the two networks were used to estimate the degree of similarities of two networks (S1 File).

Classical centrality measures were used to classify node importance while global measures were used to compare the network. In/out degree, betweenness, in/out closeness were used to characterize epidemiologically the nodes, providing information about the exposure, the risk of getting infected and infecting others nodes (S1 table in S1 File) [60]. Comparisons between centrality distributions in the two networks were done and the Kendall τ test was used to assess differences them. At global level graph density, diameter and mean distance, heterogeneity factors were used to characterize networks [60]. To assess the possible extension of outbreaks, connectivity analysis was performed, identifying the number and the size of the strong and weak connected components. Finally, a resilience study of the networks was performed to assess the impact of containment measures. Information about the measures used and the characteristics of the network can be found in (S1 File).

Community detection analysis was done to identify groups of LGAs that preferentially exchange among them. Several algorithms were tested, among them Edge Betweenness, fast-greedy, Info-Map, Louvain, Walk-trap [61,62]. The ones providing the partition in communities with modularity larger than 0.3 were retained and compared among them and the best community partition chosen among them [1]. All the analysis were conducted using R version 4.3.2, the packages igraph version 2.0.3 [58] for network analysis and visualization, ggplot version 3.5.1 [63] for visualization and SNA [64] for network analysis. Maps were created using QGIS version 3.26.2 [65].

#### Ethical approval.

Ethical clearance for the study, including the market surveys and FGDs, was obtained from the National Veterinary Research Institute Committee on Animal Use and Care, with approval number: NVRI/ AEC/02/113A/22.

## Results

Altogether, 10 markets in 10 districts were surveyed, covering 10 LGAs within three states: 2 LGAs in Bauchi, 2 LGAs in Kano and 6 LGAs in Plateau. The markets visited in Plateau include: Bukuru market in Jos South LGA, Yanshanu market in Jos North LGA, Yelwa market in Shendam LGA, Wase market in Wase LGA, Deomak market in Quaanpan LGA and Tutum market in Kanam LGA. The markets visited in Bauchi include: Gadamaiwa market in Ningi LGA and Alkaleri market in Alkaleri LGA. The markets visited in Kano include: Getso market in Gwarzo and Kara market in Wudil LGA.

### Actors involved in small ruminant movements

Among the 1065 respondents of the market survey, 89% (n = 948) were traders, while the remaining respondents were livestock owners and farmers 5.4% (n = 58), butchers 3.6% (n = 38) and transporters 2% (n = 21). Except for livestock owners, most of the actors were at the market for trading animals during market day (i.e., buying animals from a vendor to sell it at a higher price during the same day), whilst for livestock owners the main reason was for selling animals.

Concerning the market visit frequency, the majority of the respondents 40.3% (n = 429) declared to go to the market “once a week” followed by “two to three times a week” 25.4% (n = 270), “four to six times” 18.7% (n = 199) in a week and few visited “daily” 15.4% (n = 164). Only 0.2% of the respondents (n = 2) declared visiting the market just during festivities.

Participants in the FGDs were equally divided between transhumant herders and market operators. More than two thirds of the movements described were provided by market actors (n = 352), and the rest by the transhumant. Independently of the participant group, the majority 62% (n = 322) out of 517 total movements were done for the purpose of buying small ruminants.

A total of 36,798 small ruminants were moved by the respondents during the survey period of April 2022 and September 2022. Out of these 52% (n = 18,504) were goats and 48% (n = 18,286) were sheep. An average of about 23 sheep per herd of 27 goats per herd was estimated, with the maximum number for goats and sheep being 450 and 600 respectively.

The majority of the animals, 98% (n = 36,062), were moved between markets, while very few animals were sold or bought by private livestock owners, 0.5% (n = 184), 0.4% (n = 147), respectively. The remaining 1.1% (n = 405) involved movements towards slaughterhouses and other farms than the owner’s.

### Description of the market, FGDs and the combined small animal ruminant movement networks

The data collected provided a snapshot of the mobility pattern in the specific period of the year.

A total of 355 movements among 235 villages in 27 different states have been reported by respondents during the market survey. Respondents in the FGDs indicated a larger number of movements, 410, among 258 locations in 27 states. Among the 27 states identified by the participants in the two activities, 23 were in common. The geographical distances between origin and destination of the movements was estimated. In both cases the distance distribution was right-skewed (S2 File), indicating the presence of a large number of short-range movements, together with few long-range ones. Distances ranged from very few kms (2 for the market network and 3 for FGD network) to movement between locations thousand km apart (1060 km in both cases) with median distances of around 260 km in both cases. When both networks were combined, small animal movements included 31 out of the 37 states in Nigeria, thus indicating the importance of the market survey area for the flows of animals in all Nigeria. In the combined network there were 191 out of 774 LGAs, compared to FGD (156 out of 774 LGAs) and market networks (105 out of 774 LGAs) and 66 out of 774 LGAs in common indicating a low overlap value between the two networks (Table 1).

**Table 1:** Number and percentage in the Lidiski area (in parentheses) of states, LGAs, districts and villages from the market survey, FGDs and when combined (market and FGD) of small ruminant movements. In parentheses the percentage of states, LGAs, districts and villages that were in the Lidiski area.

	Market network (%)	FGD network (%)	Combined network (%)
States	27 (11.1)	27 (11.1)	31 (9.1)
LGA	105 (39.6)	156 (31.4)	191 (29.2)
Districts	149 (51.0)	209 (39.5)	291 (39.8)
Villages	235 (61.4)	258 (46.1)	422 (51.7)

### Direction of the small animal movements

Respondents in the market survey indicated a lower number of movements (355) compared to those of the FGD survey (410). The distribution among the different types of movements (Internal survey area, Incoming; Outgoing; external) was significantly different between the respondents in the market network and the respondents in the FGD network (Chi-squared = 66.5, p-value<0.001).

In both cases the majority of the small animal movements reported were located within the three states of the Lidiski Project (Internal survey area in Table 2), however respondents of FGDs identify a larger fraction of movements into the survey area from outside (Incoming in Table 2) compared to the market respondents. Conversely, market respondents indicated a larger fraction of movements from survey areas towards external ones (Outgoing in Table 2). Moreover, a very small fraction of the respondents identified the market as an intermediate stop between two villages in areas outside the survey area (external) in Table 2*.*

**Table 2:** Number and percentage (in parentheses) of small animal movements between villages as reported during the market survey activities, FGDs and when combined (market and FGDs). Internal survey area, both origin and destination were in the survey area; Incoming, only destination was inside the survey area; Outgoing, only origin was inside the survey area; External, none was inside the survey area.

	Market network (%)	FGD network (%)	Combined network (%)
Internal to Lidiski area	202 (56.9%)	196 (47.8%)	386 (51.7%)
Incoming	10 (2.8%)	96 (23.4%)	104 (13.9%)
Outgoing	136 (38.3%)	118 (28.8%)	250 (33.5%)
External	7 (2.0%)	0	7 (0.9%)
Total	355 (100%)	410 (100%)	747 (100%)

When considering the type of actors, we found that there was a significant association with them with respect to the type of movements inside or outside the survey areas (Fisher test, p-value <0.01). S2 table in S1 File.

In particular, for all the respondents of the market survey, except for the transporter, animal movements were made to intra survey area locations, while for transporters the majority of the movements were made towards or outside survey areas. Moreover, only traders reported movements from outside the three states. In the case of the FGDs, significant difference in the distribution of movement between respondents was found (Fisher test, p-value <0.001). While both transhumance and market operators indicated mostly movements inside the survey areas, market operators indicated a much larger movement from and to survey areas than the transhumant herders. Market survey respondents indicated that the fraction of movements inside the Lidiski area and outside were different if made to sell, buy or trade animals (Fisher test, p-value <0.01), whilst no significant differences were found among FGD participants (p- value >0.3).

### Network analysis

The majority of recorded small animal movements, 89.6% (318 out of 355) in the market, 95.4% (391 out of 410) in the FGDs and 93.1% (695 out of 747) in the combined networks, occurred between different districts within LGAs located in different states. Very few movements, 10.4%, 4.6% and 6.9% (37 out of 355, 19 out of 410 and 52 out of 747 movements) respectively in the market, FGD and combined networks were recorded between districts within the same LGA (Table 3).

**Table 3:** Movements among the same states, LGAs and districts in the three networks versus movements among different states, LGAs and districts in the three networks.

	Market		FGD		Combined	
	Movements in the same administrative unit	Movements in the different administrative unit	Movements in the same administrative unit	Movements in the different administrative unit	Movements in the same administrative unit	Movements in the different administrative unit
State	166	189	149	261	306	441
LGA	56	299	53	357	103	644
District	37	318	19	391	52	695

**Fig 2:**
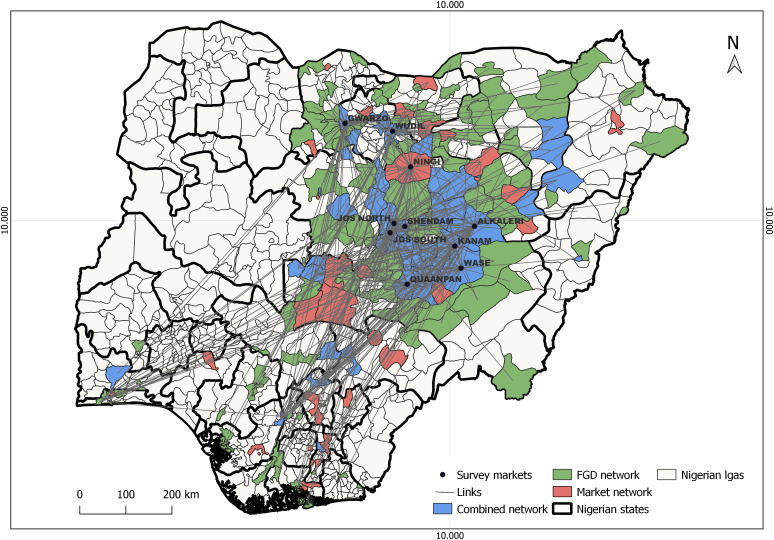
Map of the combined market and FGD network showing the LGAs where they overlap and the movement of small ruminants in Nigeria. The LGAs in red show areas of small ruminant movements collected in the market survey, the LGAs in green show areas of small ruminant movements collected in the FGD survey, the LGAs in blue show the overlapping LGAs between both the market network and the FGD network. The lines show the movement links and the black points showed the survey markets.

The combined network corresponds to the union of the two (Fig 2).

Market and FGD networks contained a large number of LGAs representing respectively 14% and 20% of all the (774) LGAs in Nigeria. The LGAs in the three survey states represented 39.6% (42 LGAs) and 31.4% (49 LGAs) of the LGAs involved in the market and FGD networks respectively. In the market and FGD networks, movements involved 32 and 25 LGAs out of the 81 LGAs of the survey area respectively.

FGD respondents identified a larger number of links but networks still were quite sparse (density between 1 and 2%). An analysis of similarity between the two networks showed a low degree of overlap both at node (Jaccard index 0.33) and link level (Jaccard index 0.13) with only 66 nodes and 61 edges in common respectively. This indicates that respondents identified different axes of movements. When combined together the fraction of LGA involved in the movements rises to almost 25% (191/774) of Nigeria LGAs.

The networks showed a low clustering coefficient (between 1 and 2%) and a small diameter [5], indicating a small world phenomenon (Table 3). Overall, the three networks were weakly connected with a single large strongly connected component (LSCC) existing in the three networks. In particular, in the combined network, almost one fourth of the nodes (n = 50) belonged to the LSCC, which was larger than the size of the two composing networks taken separately (22 nodes for market, 23 for the FGD, respectively).

Furthermore, for all the three networks, the LSCC was multi-state (5 states for both market and FGD networks, and 9 for the combined one). Besides the survey states, the LSCC for the market network contains LGAS of Yobe and Jigawa whilst the FGD ones includes LGAs from Kaduna and Nasarawa.

In addition, the combined network LSCC contains LGAs from all the aforementioned states plus LGAs from Adamawa and Borno. Moreover, in the combined network not only more states were part of the LSCC, but in each state the number of LGAS members increased more than considering the two composing networks separately. All the networks showed a high degree of in/out degree heterogeneity (Table 4), a signal of presence of few hubs in the network that could have been connected to many of the nodes.

**Table 4:** Global network characteristics. Each row corresponds to a specific global measure. Each column corresponds to the market, the FGD or the combined (market+FGD) network.

Property	Market Network	FGD Network	Combined Network
Nodes	106	156	196
Edges	211	301	451
Density	0.018958	0.01245	0.01180
Av Clust Coefficient	0.13914	0.09179	0.10985
Diameter	6	6	5
Mean Distance	3.15012	3.14248	2.97495
Weak Connected	True	True	True
Weak Component	1	1	1
Max Size	106	156	196
Strong Component	84	133	147
Max Size	23	22	50
Indegree Heterogeneity	2.3213	6.5447	5.11389
Outdegree Heterogeneity	8.34029	5.25332	8.69469
Small-worldness	7.09794	7.80858	9.40565

#### Resilience, network connectivity and frequency of movements.

**Fig 3:**
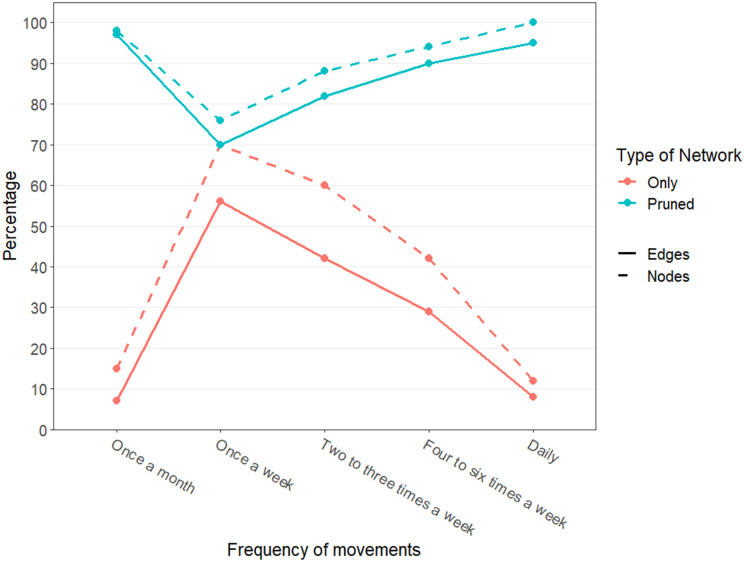
Frequency of movements and percentage of nodes in both pruned and unpruned market network (Supplementary material).

Fig 3 shows the results of the resilience analyses, where the “Only” curves correspond to the characteristics of the former network, and “Pruned” to the second one, dashed line corresponds to percentage of total nodes present in the networks, and solid line the percentage of edge.

We noticed that daily movements or extremely rare movements (once a month) contributed around 10% of LGAs and contributed to less than 10% of the links. Weekly movements involved almost 75% of the links identified by the respondents while the other two involved 60% (4–6 times) and 50% (2–3 times) of the LGAs. Independently of the frequency, all the networks were connected but their sizes varied. They exhibited a high degree of heterogeneity, in particular the 4–6 times a week one indicated the presence of a possible super-spreader (out degree heterogeneity around 10) (S3 table in S1 File).

When considering the pruned network, we noticed that many nodes (between 78% and 99%) and links (between 90% and 99%) were still in the network and similarly to the previous case, all the networks were connected. The diameter of the networks after pruning links remained constant except when monthly movements were removed, indicating the elimination of a shortcut (S4 table in S1 File). Irrespective of the frequency of movements to the LGAs, there was still a possibility of disease spreading to many other LGAs in the network.

#### Communities.

**Fig 4:**
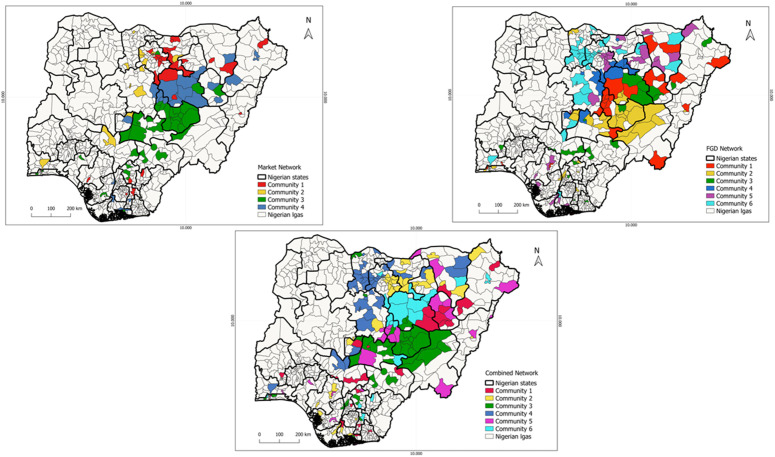
LGA communities in market network, FGD network and combined small ruminants movement network.

Five algorithm types for detecting communities have been applied, and the best community partition chosen by the modularity value. The modularity was checked to be higher than 0.3 for all the algorithms. We checked the overlap between the different algorithms using the rand index and selected the partition with the highest rand index and similar partition. The Fast greedy had the highest similarity across the algorithms hence this partition was chosen. All the communities are multi-states which can increase the risk of spread of PPR from one state to the other (Fig 4).

The communities partition from the different networks were compared to estimate the degree of overlap, if any. The rand indexes of the composing network and the combined one were quite high (0.80 for the market, and 0.94 for the FGD) indicating that partitions were similar, and the combined network one could be considered as aggregation of communities of the composing network.

**Fig 5:**
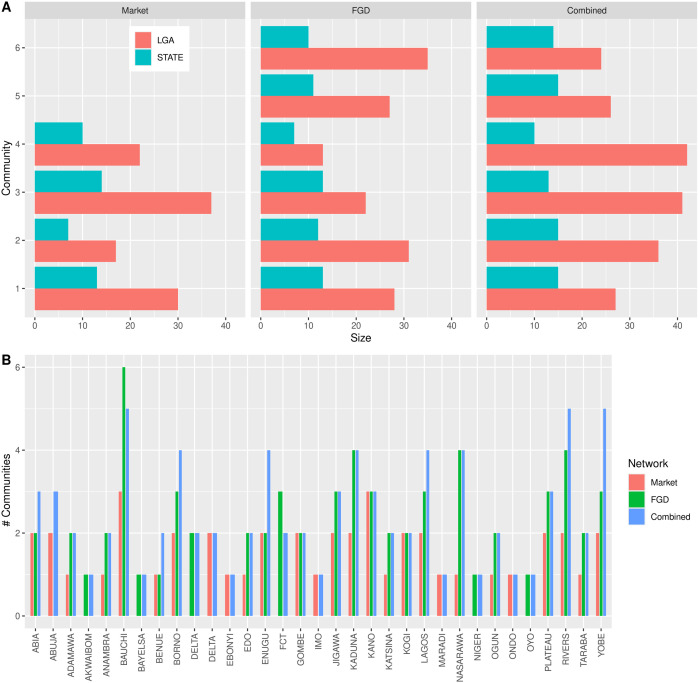
Histograms showing the distribution of communities among states in the three networks. A) Number of LGAs (red) and states (blue) in each community. Each panel corresponded to a different network. B) The number of different communities the LGAs in each state belonged to. Different colors corresponded to different networks.

Four communities were found in the market network while 6 communities were found in both the FGD network and the combined network (Fig 4). The distribution of communities’ sizes (the number of LGAs members of a community) showed the existence of at least two communities with more than 30 members. Independently of the sizes, all the communities are multistate (Figs 4 and 5). Moreover, in most of the cases LGAs of each state were distributed among different communities.

Fig 5A shows the number of states and LGAs in each community for each considered network. Fig 5B shows the total number of communities that the LGAs of a state belong to. Both figures indicated that communities were multistate (i.e., members are LGAs from different states) and that a state’s LGAs belonged to different communities (i.e., states are multi-communities). This was particularly true for the FGD network, where almost all the states had LGAs that belonged to different communities, and this number increased further when the two networks were combined, except for Bauchi and the FCT states.

### In the market network, none of the states belonged to all communities, whilst Bauchi LGA appeared in all communities

#### Centrality measures.

**Fig 6:**
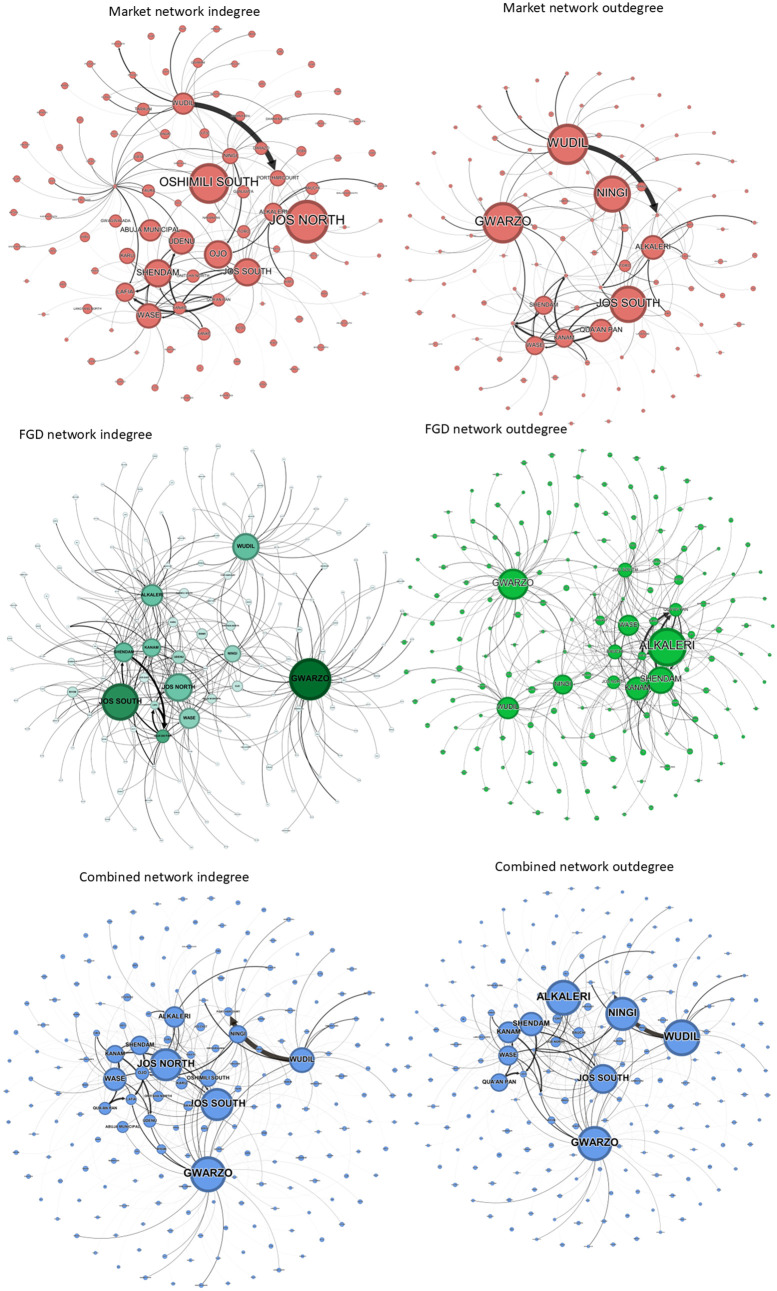
Market network, FGD network and combined network sorted by indegree and outdegree. The node size for both the indegree and outdegree corresponds to the LGAs with the largest indegree and largest outdegree, respectively.

All the networks showed a high degree of indegree (indeg) heterogeneity. κ value for the combined network (κ  = 8.27) was higher than both the market network (κ  = 2.35) and the FGD network (κ  = 6.545) as seen in Table 4. Gwarzo had the highest indegree (indeg = 31) (Fig 6) in both the FGD and combined network respectively (indeg = 31) while analysis of the market network indicated Jos north (indeg = 14) having the largest indegree (Fig 6).

The indegree distributions of the FGD and market network were different (Kendall τ test = 0.122, p-value = 0.052). Similarly to the previous case, also for the outdegree (outdeg), all the networks exhibit strong heterogeneity. Coefficient κ of the combined network (κ = 8.27) was higher than that of the market network (κ = 7.93) and that of the FGD network (κ = 5.253). In the combined network, Alkaleri was the most (out) connected node (outdeg = 38). However, when we considered the networks separately, Alkaleri (outdeg = 28) in the FGD network and Gwarzo (outdeg = 27), and Wudil (outdeg = 27) in the market network were the most connected (Fig 6). We notice that Wudil’s high outdegree was the combination of information from the two activities. The outdegree distributions of the market network and the FGD network differed significantly (Kendall τ test = 0.409, p-value <0.0001).

The nodes with the highest betweenness (betw) are Jos North LGA (betw = 0.201) in the combined network Jos South LGA (betw = 0.0917) in the market network, and Alkaleri (betw = 0.1329) in the FGD network, as seen. Similarly, the fact of combining the information of the two sources changes the role of nodes in the networks.

The incloseness (incloss) centrality was almost similar in the combined and FGD networks. The highest incloseness value was seen in Gwarzo (incloss = 0.0005) in the combined network, Shanono (incloss = 0.059) in the market network and Jos South (incloss = 0.001) in the FGD network. The LGAs with the highest outclosesness was Kaltungo (incloss = 0.077) in the market network, while that in the FGD network was Gwarzo (incloss = 0.005).

There were few clustering nodes overall, but the market network had more cluster nodes, with a value closer to 1 than the FGD network.

#### Identification of potential super-spreaders and super-receivers.

**Fig 7:**
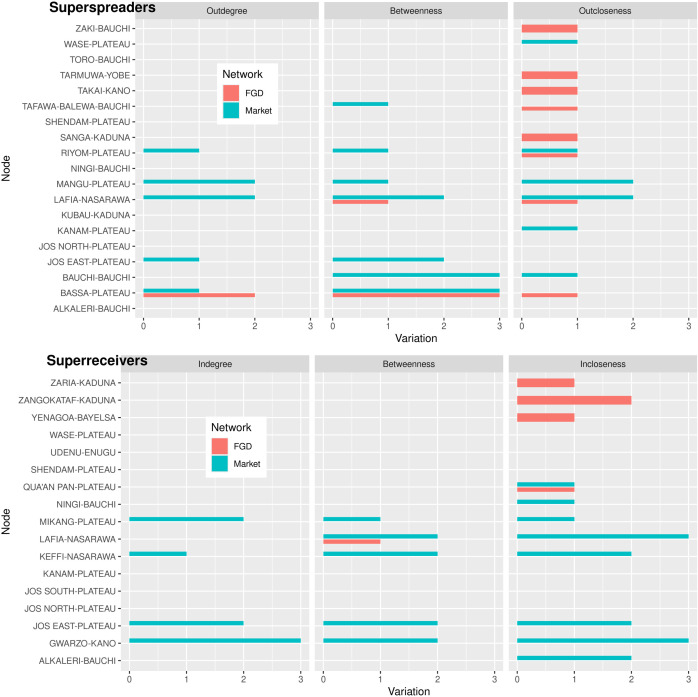
Bar plot showing the variation in quantile for potential super-spreaders and super- receivers. The height of the bar corresponds to the variation in the centrality measure quartiles classes. Color corresponds to the network.

Fig 7 shows the list of potential super-spreaders and super-receivers in the combined network, the length of the bar accounts for the change in quartile position, while color corresponds to the original network. For most of the super-receivers the combination of information resulted in a large increase in quantile positions. There were 19 potential super-spreaders, 5 were super-spreaders in the market network, and 11 in the FGD one. Similarly, among the 17 potential super- receivers, 6 were super-receivers of the market network, and 12 in the FGD one (Fig 7).

#### Resilience and cutting points.

**Fig 8:**
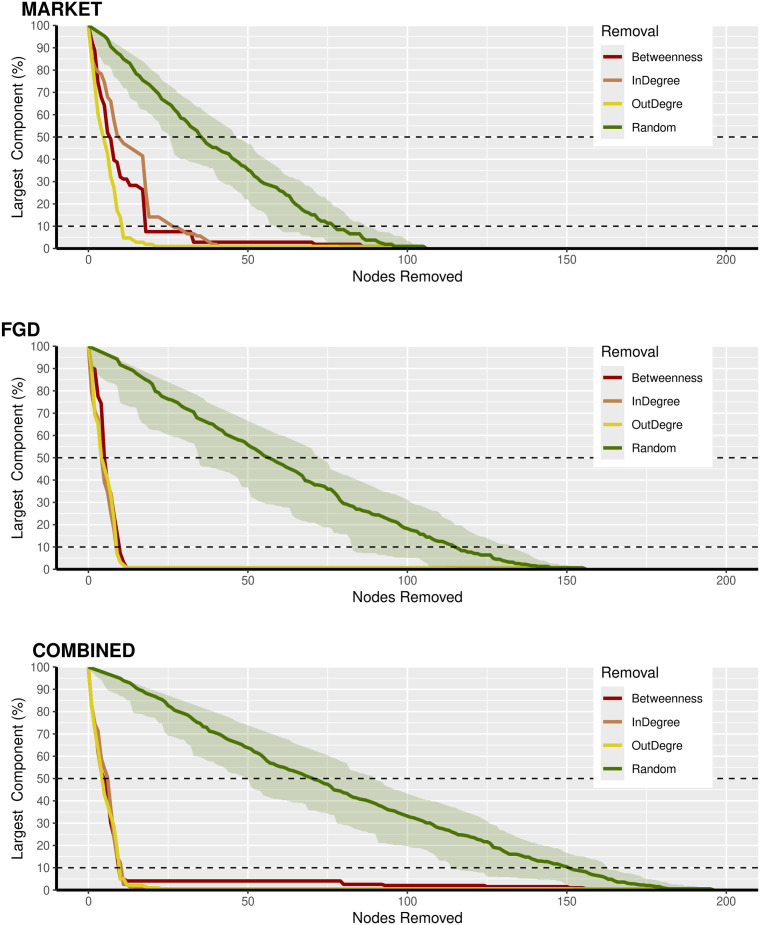
Random removal and targeted removal of nodes for each network row, the size of the GCC is a fraction of the total original network after removal of a node. In green, random removal (shaded area 95% CI after 50 simulations). Targeted removal (Red based on betweenness of the nodes; Orange based on Indegree; Yellow based on Outdegree).

We tested the network’s resilience by removing nodes and their links. We considered both random failures (i.e., removing nodes randomly, green area in the Fig 8) and targeted ones based on three criteria (Outdegree, Indegree, Betweenness).

All the three networks were resilient against random attacks. Market network appeared particularly fragile to removal based on outdegree. After 10 nodes have been removed the greatest connected component (GCC) size was reduced to 10% of the original one (S5 table in S1 File). For the FGD and combined ones, no difference between removal criteria have been found and, similarly to the market case, the removal of the top 10 nodes (9 for the FGD network) shattered the network to small sub-networks, whose larger size corresponds to 10% of the nodes.

Eight of these cut points were common to all the three networks, albeit with a different importance, and correspond to LGAs where survey was conducted (except Qua’an Pam in Plateau state and Jos North in Plateau). In the market network, the LGA of Toro in Bauchi, although not surveyed, appeared as a cut-points more important than Jos North. The impact of LGAs’ removal could be estimated by the reduction in size of the GCC after the removal: more nodes were disconnected after the removal, the more important the LGA. The importance of the nodes varied among the network. In the market network Wudil and Ningi were the most important nodes. Gwarzo appeared to be the most important node in the removal of FGD and combined networks. We noticed that Jos North despite being the one with lowest indegree, was the second most important LGAs.

## Discussion

The knowledge of the animal movement patterns and trade routes among various populations of hosts is particularly necessary because it represents the critical driver of spread for many TADs, defining the pathways along which transmission can occur. Understanding small ruminant movement patterns will support the improvement of specific surveillance and control strategies for major TADs like PPR [63].

This study was the first of its kind to describe the network of small ruminant movements in three selected states in Northern Nigeria (Bauchi, Kano, Plateau) using a mixed approach for collecting data. Gathering livestock movement data is extremely challenging, primarily due to the difficulty of designing methods for collecting data that ensure adequate coverage. This survey typically uses existing national monitoring systems. However, in areas lacking such systems or where market access is difficult, new methods must be developed to gather data from a representative sample of markets. Because of this an approach involving a survey and participative FGDs has been developed to retrieve the maximum of information. Data collected in this way can be used to inform more advanced mathematical models to better predict movements [66–68]. This approach has allowed us to have a clear view of mobility patterns, without doing snowball sampling of markets, in areas presenting logistical difficulties. Combining the two methods of data collection strengthened, complemented and completed our understanding of livestock (small ruminant specifically) commercial mobility in Nigeria. This integration bridges data gaps, providing a more complete understanding of why animals move, not just where, improving network analysis accuracy”. Indeed, the analysis shows that by surveying the three states targeted in this study, we covered an area at the crossroads of movements among almost all the Nigerian states (31 over 37) whilst, when taken singularly, each network contains LGAS from just 27 states.

Respondents of the market survey were traders, transporters, livestock owners and butchers, whilst for FGD were market operators and transhumant herders. Except for transporters, all the respondents provided information mostly about movements within the survey area. On the other hand, transporters provided information about movements originating in the survey areas towards other Nigerian states. This is because a large number of sellers usually pay the transporters to move their animals from these survey areas to other states for sales. Similarly, responses provided by FGD were different according to the type of respondent, with market operators providing more information about outgoing movement compared to transhumant herders.

Focusing on the market surveys, results showed that markets are mostly a place for trade where animals are sold and bought by traders during the same day, whilst owners and butchers tend to either sell or buy animals respectively. Epidemiological surveys conducted in the some of the study’s markets, showed that diseases like PPR, FMD, Sheep and Goat Pox, were circulating among traded animals, further reinforcing the belief that markets are hotspot for the spread of disease and the need to conduct activities to map the movements [69]. Most of the respondents were trading both sheep and goats at the same time although a slightly larger number of sheep are sold and bought compared to goats. This slight difference could be related to the period of the survey (April 2021 to June 2022) close to “Eid-el-Kabir”, a religious festivity, during which Muslims are known to buy and slaughter sheep to celebrate [1,25,48]. This seemed to be more prominent in the northern region which has a higher concentration of Muslims than in the southern region of Nigeria.

### Animal mobility and disease spread

All the networks appear to be sparse (low link density) are sparsely connected and highly heterogeneous both in the indegree and outdegree, indicating the presence of hubs, but also a hierarchy in the supply chain. Whilst, fewer connections mean fewer opportunities for infection to jump directly between LGAs, the presence of hubs, could facilitate and speed up the diffusion of pathogens. These networks’ low densities (sparse connections) limit the spread of diseases. In particular, the large outdegree heterogeneity could indicate the presence of a few markets serving several consumption areas that could facilitate the spread of disease**.**

All the networks were connected and compact (i.e., small diameter), thus in a few steps, diseases can potentially spread to all the other nodes in the networks.

LSCC usually represents a lower bound for outbreak extension since its presence could favor the circulation of a pathogen among its members. When networks are combined, the size of the LSCC becomes more than twice larger, indicating that new connections are formed and, hinting that if an epidemic occurs it can reach more LGAs than if only one type of data is considered. Because of this, it becomes necessary for Veterinary Services to collect information of mobility from different sources to have a better estimate of epidemics extent and better identify a set of areas that could maintain the virus in circulation (nodes in the LSCC). Moreover, the market network is also resilient against elimination of movements with specific frequency and almost 70% of the LGAs in the network can still be reached when only weekly movements are considered. In all the cases the LSCC includes areas from different states, thus increasing the risk that disease cannot be contained within the border of a state but needs a harmonized translation effort. All the networks show a small-world effect, indicating the existence of clusters in the network that could facilitate transmission among neighbors, but also shortcuts that could speed the spread of pathogens in farthest areas of the networks (and Nigeria) [70].

Four to six communities have been identified among the three networks and, independently of the network considered, all appear to be multi-state. Communities are tightly connected sets of nodes in the network whose presence is a sign that underlying dynamics are taking place favoring interactions among specific subsets of nodes. Recently it has been argued that PPR genetic clusters are strongly related to livestock mobility network communities and that belonging to a community increases the risk of being infected [70,71]. Moreover, due to the fact that LGAs of each state belong to different communities, studying mobility patterns in other states would help to identify bridge areas that act as connections among communities.

The three states in the Lidiski project were found to be in more than one community. This was also true for several other states. This suggested that in case of an outbreak, the disease had more than one way to enter the states. Study of the genetic diversity of PPR virus in Nigeria supports this hypothesis of high connectivity across all Nigerian states [56,57].

#### Implications for disease management.

The study emphasizes the need for more animal movement surveys in Nigeria. This data would allow for comparison with existing networks and identification of high-risk areas for diseases like PPR. Such information is crucial for efficient disease surveillance and control, particularly in resource-limited settings. The methodology presents some advantages for a data-limited environment to identify larger networks. By combining the information from the two data sources it appeared that the areas of survey are at the crossroad of movements within the vast majority of states in Nigeria (31 over 36). Overall, the study emphasizes the importance of network centrality and global measures in understanding disease spread within animal movement networks. This is in phase with recommended strategies for the controls of TADs, notably within the frame of the global eradication campaign for PPR led by FAO and WOAH [67], to improve surveillance systems using risk-based approaches to optimize resources and personnel. Identifying critical LGAs based on in-degree, out-degree, betweenness, closeness and communities in addition to testing the network for resilience allows for targeted interventions and control strategies to minimize disease outbreaks. Based on centrality measure analysis of very few nodes, 17 corresponding to super-receivers appears to be good candidates for being elements of a surveillance network. Among those, only 6 were part of the 10 surveyed markets, while the others were locations identified by the respondents. Moreover, combining the information of the two surveys, several locations were promoted to the rank of super-receivers, highlighting once more, the importance of collecting detailed information on movements.

The resilience analysis has shown that the control intervention in just markets in 10 LGAS could efficiently stop the diffusion of the disease. Control measures could involve prioritizing vaccination of animals in the abovementioned LGAs (Gwarzo and Wudil in Kano; Alkaleri and Ningi in Bauch; Jos South, Jos North, Shendam Wase Kanam Qua’an Pan in Plateau) by vaccinating (and temporary holding resting) animals at the markets in these LGAs, or banning unvaccinated animal movements from this area. Despite the support that modeling could provide, several other factors should be taken into account to make these measures sustainable, like the economic feasibility of restraining movements and the logistic efforts of vaccination.

Finally, community structures indicate strong and frequent relations among LGA of different states, requiring a harmonized plan of control among states members of the same community. Community partition could be used to delineate areas to improve targeted surveillance and control measures. Coordination among the different states and Veterinarian services would be required. Moreover, detecting “overlapping LGAs”, i.e., those that could be part of different communities, could help identify possible crossroads of different transmission patterns which may be crucial for both epizootic and endemic disease control efforts [48].

#### Limitations of the study.

The study collected data from ten strategically chosen districts – all identified as having the highest number of incoming movements (in-degree) or outgoing movements (out-degree). While focusing on these central districts is valuable, it raises concerns about selection bias. By excluding less connected districts, the study might underestimate their role in disease spread within the network.

Despite the amount of information retrieved combining the two networks, one of the limitations of this approach resides in the validation and cross reference of FGD data. To overcome this limit, market surveys should be conducted in FGD identified areas to confirm the presence of movements. Data were collected in a specific period of the year, however, due to religious festivity, livestock demographic events, seasonality and husbandry choices, network structure could change abruptly along the year. Regular surveys at markets could fill this gap, as well as restructuring FGD questionnaires to include detailed information about structural changes.

All the centrality and global measures have been considered as proxy to estimate the extension of a potential epidemic and the role of the nodes. However, the spread of diseases depends not only on the structure of the network but also on the characteristics of the diseases itself. Because of this, numerical simulation is needed to confirm the conclusions of this work, to identify sentinel nodes and to assess the impact of control measures. Finally, mobility is one of the risk factors for the spread of small ruminant transboundary diseases and needs to be combined with other risk factors to assess the spatial risk for disease introduction.

From a research point of view, future works should be directed towards:

- Validating the FGD responses. Locations identified by FGD participants, that weren’t indicated in market survey, could be used a new market survey locations to collect further data and estimated the flows of animals
- Confirming seasonality of animal movement. As today, the reconstructed network represents a one-shot picture of livestock mobility patterns. However, due to religious festivities and social events, economic environmental factors, sanitary situation, the structure of movements and flows can change along the year. By repeatedly surveying markets, possible regularities of movements could be found and relate to socio-economic and environmental factors. This in turn would help in modeling the dynamics of livestock mobility and provide useful indicators for risk of disease introduction.
- Investigating short scale movements. In this work, most of the data collected concerned movements between markets that imply long range movement. However, to provide a more accurate description of mobility patterns and improve risk assessment, movements between farms and markets should be included. In this case markets indicated in the survey could be used as new survey areas.

## Conclusion

This study, focused on small ruminant movement in northern Nigeria, addressing a broader need for improved methodologies in animal movement epidemiology. Many regions, like northern Nigeria, struggle with incomplete data and informal movement patterns due to a lack of formal animal identification systems and demand the development of new tools for filling gaps. The combination of market survey and qualitative approaches provide complementary representation of mobility patterns and allow to identify relations and structures otherwise not visible when analyzing the data separately. The two approaches have provided a wealth of information about mobility patterns that could inform competent bodies for animal health and surveillance. The analysis of movement patterns identified that the three states of the Lidiski Project were at the crossroads of movements among locations in almost all Nigerian states. In particular, the data collected showed that a large, single network exists among locations in Nigeria, which could facilitate the spread of PPR and similar TADs throughout Nigeria in a matter of weeks. Moreover, the analysis helps identify other important areas, like the Toro LGA in Bauchi, that were not included among the surveyed areas. These findings emphasize the need for a harmonized surveillance and communication plan among different veterinary services to improve the monitoring and control of TADs.

## Supporting information

S1 File Network analysis. (DOCX)

S2 File Distribution of distances between locations in the Market and FGD network. (TIFF)

S3 File Market survey questionnaire. (DOCX)

S4 File FGD guide. (DOCX)
