# Supplementary material for: Combining market surveys and participative approaches to map small ruminant mobility in three selected states in northern Nigeria
Source: PLoS One. 2025 Sep 2;20(9):e0311030. doi: 10.1371/journal.pone.0311030 (PMC12404370; doi:10.1371/journal.pone.0311030)
Supplement: S1 File — (DOCX) [file pone.0311030.s001.docx]

**S1. NETWORK ANALYSIS**

Given two sets of elements A and B the Jaccard index was estimated as:

$J(A,B)=\frac{|A\cap B|}{|A\cup B|}$

where || indicates the dimension of the set, $A\cap B$ is the intersection of the two sets (i.e. the common elements) and $A\cup B$ is the union of the two sets (i.e. all the distinct elements). The jaccard index varies between 0 (no similarity) and 1 (identical).

Classical centrality measures (S2 table) were used to classify node importance. Centrality measures were needed to determine the potential roles of different nodes (LGAs) in the spread of diseases through animal mobility. In/out degree, betweenness, in/out closeness were used to characterize epidemiologically the nodes, providing information about the exposure, the risk of getting infected and infecting others nodes (S2 table). Comparisons between centrality distributions in the two networks were done and the Kendall 𝜏 test was used to assess differences them.

Global measures were used to characterize the networks as a whole and to compare networks. In particular: graph density, diameter and mean distance (1), heterogeneity factors and parameters were used. Graph density, ratio between existing links and all potential links that can be formed in a network, was used to assess whether a network was fully connected or sparse. The diameter (2) was used for the rapidness of the disease transmission and the presence of shortcuts. The heterogeneity parameter $\kappa$ provides information about the skewness of the distribution and the presence of hubs in the network.

$\kappa_{i,o}=\frac{<k_{i,o}^{2}>}{<k_{i,o}>^{2}}$

where $k_{i,o}$ indicates in/out degree and <> indicates average value. For homogeneous distribution the $\kappa_{i,o}\simeq1$ while heavy tail distribution $\kappa_{i,o}>$>1.

To assess the possible extension of outbreaks, connectivity analysis was performed, identifying the number and the size of the strong and weak connected components. For the market network only, since information about the frequency of the movements were available, we compared connectedness of the static representation against networks pruned of links of specific frequency (pruned network) and network formed of just links of specific frequency (unpruned network). This would provide information about their role on the pathogen diffusion.

To assess the possible impact of containment measures, like movement interdiction from LGAs and/or vaccination in specific areas, we tested the resilience of the network under random and targeted removal (1–3). We tested the network's resilience by removing nodes and their links to determine which of the centrality measures could be primarily recommended for managing outbreaks of these small ruminant diseases. Two methods of node removal were employed. In the random removal case, nodes were removed randomly, while in the case of targeted removal, nodes were ordered based on (Outdegree, Indegree, Betweenness) centrality measure and removed subsequently from the highest value to lowest. For the market network only, we considered two additional analyses. First, we focused on networks formed by movements with a specific frequency, then we considered networks pruned of links with specific frequency.

At each node removal, size of the Greatest Connected Component was estimated, together with importance of the node expressed as the number of nodes disconnected by the network.

In this work, we define as potential super-spreaders those nodes that have not only the largest outdegree, but also betweenness and outcloseness (4,5). Betweenness has been chosen as a parameter for centrality and the possibility that the node could be part of propagation path of a pathogen, while outcloseness has been used as a proxy to the extension of nodes that can be reached in the shortest amount of time by the potential super-spreader: the higher the faster nodes can be infected. In a similar way, super-receivers are nodes that are most likely to be reached. We use them as proxies for identifying potential super-receivers of the (largest) indegree, betweenness (largest) and incloseness. To assess the impact of combining the networks on the potential super-spreaders and super-receivers, we calculate for each potential super-spreader/super-receiver the variation in quantile class.

**Tables of network analysis results**

*S1 table. Network indicators, definitions and their epidemiological interpretation (6)*

|  | Indicator | Definition | Interpretation |
| --- | --- | --- | --- |
| Central measures | In/Out Degree | Number of Incoming / Outgoing links between nodes | Incoming movement may contribute to disease introduction. Outgoing movements may contribute to disease dissemination. |
| Central measures | Betweenness centrality | For a node, it is the number of shortest paths using this node to link a pair of other nodes of the network. | Identifying trade crossroads requires prioritizing surveillance efforts. |
| Central measures | Clustering coefficient | For a node, probability that its linked nodes are also directly connected to each other. | Finds groups of closely linked  sites that could facilitate the spread of disease. |
| Global Measures | Mean distance | Mean distance shows the average of all the shortest path between two closest nodes while the Longest path is the path between 2 more distant nodes. | It is useful to assess diseases spread. Time to spread between nodes and through all network |

*S2 table: Distribution of movements intra- and inter-Lidiski State by type of data collection and type of respondent.*

|  | Market | | | | Fgd | |
| --- | --- | --- | --- | --- | --- | --- |
|  | Butcher | Owner | Trader | Transporter | Market Operators | Transhumance |
| Internal | 36 | 45 | 647 | 6 | 116 | 101 |
| Incoming | 0 | 0 | 24 | 0 | 77 | 27 |
| Outgoing | 1 | 10 | 279 | 17 | 102 | 27 |
| External | 0 | 1 | 8 | 0 |  |  |

*S3 table. Description of the market network characteristics showing the frequency of market visits without pruning*

| Property | Daily | Four to Six Times Weekly | Two to Three Times Weekly | Once Every Week | Once Every Month |
| --- | --- | --- | --- | --- | --- |
| Nodes | 12 | 44 | 63 | 75 | 16 |
| Edges | 16 | 61 | 90 | 118 | 14 |
| Density | 0.12121 | 0.03224 | 0.02304 | 0.02126 | 0.05833 |
| Av Clust Coefficient | 0.3125 | 0.10248 | 0.06023 | 0.09868 | 0.10714 |
| Diameter | 3 | 6 | 5 | 6 | 2 |
| Mean Distance | 1.375 | 2.49231 | 2.27666 | 2.39957 | 1.44 |
| Weak Connected | False | True | True | True | False |
| # Weak Comps | 2 | 1 | 1 | 1 | 3 |
| Max Size | 9 | 44 | 63 | 75 | 10 |
| # Strong Comp | 11 | 39 | 58 | 69 | 16 |
| Max Size Strong | 2 | 4 | 4 | 3 | 1 |
| Indegree Heterogeneity | 1.40625 | 1.35985 | 1.63333 | 2.52083 | 1.46939 |
| Outdegree Heterogeneity | 4.125 | 6.25530 | 10.60889 | 5.49411 | 3.42857 |

*S4 table. Description of the market network characteristics showing the frequency of market visits when pruned***.**

| Property | Daily | Four to Six Times Weekly | Two to Three Times Weekly | Once Every Week | Once Every Month |
| --- | --- | --- | --- | --- | --- |
| Nodes | 106 | 100 | 93 | 81 | 104 |
| Edges | 201 | 188 | 173 | 146 | 205 |
| Density | 0.01806 | 0.01899 | 0.02022 | 0.02253 | 0.01914 |
| Av Clust Coefficient | 0.12378 | 0.13142 | 0.1555 | 0.1125 | 0.13569 |
|  | 6 | 6 | 6 | 6 | 7 |
| Mean Distance | 3.00493 | 3.22074 | 2.9006 | 3.13555 | 3.25672 |
| Weak Connected | TRUE | TRUE | TRUE | TRUE | TRUE |
| # Weak Comps | 1 | 1 | 1 | 1 | 1 |
| Max Size | 106 | 100 | 93 | 81 | 104 |
| # Strong Comp | 87 | 83 | 77 | 63 | 82 |
| Max Size Strong | 18 | 18 | 16 | 19 | 23 |
| Indegree Heterogeneity | 2.27999 | 2.50113 | 2.36466 | 1.77078 | 2.25447 |
| Outdegree Heterogeneity | 8.79201 | 7.571299 | 6.86415 | 8.93751 | 8.42146 |

*S5 table.  Summary of the removal procedure.*

*For each network, the list of the LGA consecutively removed before reducing the GCC size to less than 10% (in red less than 50%) and the reduction of the GCC size after each removal (in blue the most important).*

| Market | | FGD | | Combined | |
| --- | --- | --- | --- | --- | --- |
| LGA | Size Reduction | LGA | Size Reduction | LGA | Size Reduction |
| Wudil-Kano | 15 | Alkaleri-Bauchi | 15 | Gwarzo-Kano | 34 |
| Gwarzo-Kano | 13 | Gwarzo-Kano | 33 | Wudil-Kano | 21 |
| Ningi-Bauchi | 14 | Shendam-Plateau | 6 | Alkaleri-Bauchi | 17 |
| Jos South-Plateau | 7 | Kanam-Plateau | 8 | Ningi-Bauchi | 20 |
| Alkaleri-Bauchi | 6 | Wudil-Kano | 23 | Jos South-Plateau | 20 |
| Qua'an Pan-Plateau | 6 | Ningi-Bauchi | 8 | Shendam-Plateau | 9 |
| Shendam-Plateau | 11 | Wase-Plateau | 11 | Kanam-Plateau | 10 |
| Wase-Plateau | 9 | Jos South-Plateau | 17 | Wase-Plateau | 15 |
| Kanam-Plateau | 7 | Jos North-Plateau | 24 | Qua'an Pan-Plateau | 14 |
| Toro-Bauchi | 3 |  |  | Jos North-Plateau | 26 |

**References**

1. Crucitti P, Latora V, Marchiori M, Rapisarda A. Efficiency of scale-free networks: Error and attack tolerance. Physica A: Statistical Mechanics and its Applications. 2003;320:622–42.

2. Sosa S, Sueur C, Puga-Gonzalez I. Network measures in animal social network analysis: Their strengths, limits, interpretations and uses. Methods Ecol Evol. 2021;12(1):10–21.

3. Gates MC, Woolhouse MEJ. Controlling infectious disease through the targeted manipulation of contact network structure. Epidemics. 2015;12:11–9.

4. Ciss M, Giacomini A, Diouf MN, Delabouglise A, Mesdour A, Garcia Garcia K, et al. Description of the Cattle and Small Ruminants Trade Network in Senegal and Implication for the Surveillance of Animal Diseases. Ren LZ, editor. Transbound Emerg Dis. 2023;2023:1–13.

5. Dubé C, Ribble C, Kelton D, McNab B. Introduction to network analysis and its implications for animal disease modelling. OIE Revue Scientifique et Technique. 2011;30(2):425–36.

6. Badham, Jennifer & Stocker, Rob. (2009). The impact of network clustering and assortativity on epidemic behaviour. Theoretical population biology. 77. 71-5. 10.1016/j.tpb.2009.11.003.
