# Supplementary figures and images for: Combining market surveys and participative approaches to map small ruminant mobility in three selected states in northern Nigeria
Source: PLoS One. 2025 Sep 2;20(9):e0311030. doi: 10.1371/journal.pone.0311030 (PMC12404370; doi:10.1371/journal.pone.0311030)

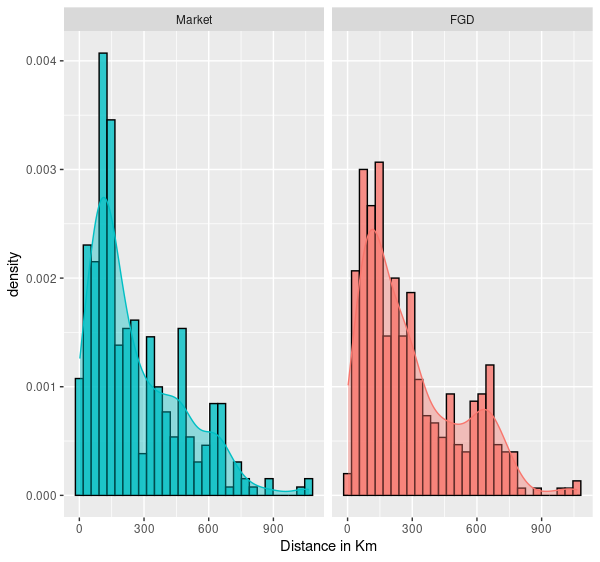

Supplement: S2 File — (TIFF) [file pone.0311030.s002.tiff]
