# Supplementary material for: Combining market surveys and participative approaches to map small ruminant mobility in three selected states in northern Nigeria
Source: PLoS One. 2025 Sep 2;20(9):e0311030. doi: 10.1371/journal.pone.0311030 (PMC12404370; doi:10.1371/journal.pone.0311030)
Supplement: S3 File — (DOCX) [file pone.0311030.s003.docx]

| **Type of Movement**  Commercial  Transhumant | | **Reason**  Buy  Sell  Buy and sell | | | | **Species**  Cattle  Goats  Sheep | | | **Type of Place**  Own Property  Market  Other Farm  Slaughterhouse | | | | **Transport**  Vehicle (motorcycle, truck, car...)  By foot | | **Actor**  Livestock Owners  Traders  Farmers  Butchers  Transporters | | | | **Frequency**  Daily  Every Month of the year  Four to six times weekly  Two to three times weekly  Once every week  Once every month | | | | |  |
| --- | --- | --- | --- | --- | --- | --- | --- | --- | --- | --- | --- | --- | --- | --- | --- | --- | --- | --- | --- | --- | --- | --- | --- | --- |
| **Type of movement** | **Date** | **Origin** | | | | | | **Destination** | | | | | | | | **Transport** | **Actor** | **Reason** | | **Frequency** | **Species** | **Herd size** | **Type of place** | |
|  |  | Country | State (Admin1) | LGA  (Admin2) | District  (Admin3) | | Village | Country | | State (Admin1) | LGA  (Admin2) | District  (Admin3) | | Village | |  |  |  | |  |  |  |  | |
|  |  |  |  |  |  | |  |  | |  |  |  | |  | |  |  |  | |  |  |  |  | |
|  |  |  |  |  |  | |  |  | |  |  |  | |  | |  |  |  | |  |  |  |  | |

**S3: MARKET SURVEY ANIMAL MOBILITY QUESTIONNAIRE**
