# Supplementary material for: Combining market surveys and participative approaches to map small ruminant mobility in three selected states in northern Nigeria
Source: PLoS One. 2025 Sep 2;20(9):e0311030. doi: 10.1371/journal.pone.0311030 (PMC12404370; doi:10.1371/journal.pone.0311030)
Supplement: S4 File — (DOCX) [file pone.0311030.s004.docx]

**S4. FOCUS GROUP DISCUSSION GUIDE FOR SELLERS AND BUYERS (TRADERS) AND TRANSPORTERS OF SHEEP AND GOAT**

**Study population:** small ruminant sellers, buyers and transporters in the market (only 10 people per market will be selected randomly) chosen after discussion with market leader

(Each livestock market visited it will be enquired, if there are records of animals sold and/or transactions in the market. If there are, permission will be sought to have access to this data). The focus group meeting will occur only during the first visit. The second visit will use the questionnaire kobo collect tool.

**SECTION1: DEMOGRAPHICS/IDENTIFICATION**

Traders will identify themselves, age, experience, and market. Consent forms will be signed or consent will be given orally. GPS will be taken. Contact information will be taken if needed to contact for further information

Date: __________________        Code_____________   GPS____________

Name of interviewer___________ State___________     LGA: _______________ _ Village _ ________________            District___________ Name of the market: ________

**SECTION 2: ECONOMIC PURPOSES AND LOCATION OF HOTSPOTS**

1.   What are the purposes of small animals brought to the market? *(rank them using proportional piling*).

2.   List the fattening centres, slaughter houses/ abattoir, watering points, feeding points in the market/LGA.

**SECTION 3: LIVESTOCK MOVEMENT**

3.   Where do most of the small ruminants come from to this market? *(Fill the following on the flip chart)*

| Name of places | State | LGA | Village | Proportion |
| --- | --- | --- | --- | --- |
|  |  |  |  |  |
|  |  |  |  |  |

4 . Where do you go to sell your small animals *(use proportional piling)? (Fill the following on the flip chart)*

| Name of places | State | LGA | Village | Proportion |
| --- | --- | --- | --- | --- |
|  |  |  |  |  |
|  |  |  |  |  |

5. When do you go to these markets to buy and sell small ruminants? *(Fill the following on the flip chart)*

| *Name  of Market* | Jan | Feb | Mar | Apr | May | June | Jul | Aug | Sep | Oct | Nov | Dec |
| --- | --- | --- | --- | --- | --- | --- | --- | --- | --- | --- | --- | --- |
|  |  |  |  |  |  |  |  |  |  |  |  |  |
|  |  |  |  |  |  |  |  |  |  |  |  |  |

6.   Why do you choose these markets to buy and sell?

| Market | Reason |
| --- | --- |
|  |  |
|  |  |

**SECTION 4: RETROSPECTIVE STUDIES**

*SEASONAL CALENDAR*

7. Seasonal calendar: How does the season affect the following factors?

Probe: In which months do you experience rainfall, dry season, more pasture, more presence of shared ponds, more animal births, more animal deaths?

- *Probe: When do you have more sheep and goat births and death proportion,*
- *When do you have the most pasture, rainfall period, animal density, animal movement?*
- *When do you have festivities? In what months do you have the most sales of sheep and goats ? In what months do you purchase the most sheep and goats?*
- *When do you normally observe PPR, when was the last outbreak of PPR in the markets, and in the LGA?*
- *When do you vaccinate your sheep and goat against PPR?*
- *When do transhumance come to you?*
- *When do you go to the transhumance?*

*(Tick as appropriate on the table below)*

|  | Jan | Feb | Mar | Apr | May | June | Jul | Aug | Sep | Oct | Nov | Dec |
| --- | --- | --- | --- | --- | --- | --- | --- | --- | --- | --- | --- | --- |
| *Rainfall* |  |  |  |  |  |  |  |  |  |  |  |  |
| *Dry season* |  |  |  |  |  |  |  |  |  |  |  |  |
| *Pasture* |  |  |  |  |  |  |  |  |  |  |  |  |
| *Ponds* |  |  |  |  |  |  |  |  |  |  |  |  |
| *Births* |  |  |  |  |  |  |  |  |  |  |  |  |
| *Deaths* |  |  |  |  |  |  |  |  |  |  |  |  |
| *Sale* |  |  |  |  |  |  |  |  |  |  |  |  |
| *Purchase* |  |  |  |  |  |  |  |  |  |  |  |  |
| *Festivities* |  |  |  |  |  |  |  |  |  |  |  |  |
| *PPR in market* |  |  |  |  |  |  |  |  |  |  |  |  |
| *PPR in LGA* |  |  |  |  |  |  |  |  |  |  |  |  |
| *Vaccination* |  |  |  |  |  |  |  |  |  |  |  |  |
| *Market prices are higher* |  |  |  |  |  |  |  |  |  |  |  |  |
| *Market prices are lower* |  |  |  |  |  |  |  |  |  |  |  |  |
| *When do transhumance come to you* |  |  |  |  |  |  |  |  |  |  |  |  |
| *When do you go to the transhumance* |  |  |  |  |  |  |  |  |  |  |  |  |

**SECTION 5: DISEASE KNOWLEDGE, PREVENTION AND CONTROL MEASURES**

8. What are the priority diseases in small ruminants? (use a disease matrix table)

*Fill the following on the flip chart)*

| DISEASE NAME | CLINICAL SIGN | SEASON | AGE | SEX | CAUSE | PREVENTION | TREATMENT |
| --- | --- | --- | --- | --- | --- | --- | --- |
|  |  |  |  |  |  |  |  |
|  |  |  |  |  |  |  |  |

*9.* Vaccination: what is their knowledge and use of vaccines, vaccine coverage for PPR and their recommendations for vaccine coverage.?

10. Where do you get your vaccines from *(rank using proportional piling*)?

11. What measures do you have to prevent and control the introduction of disease from sick animals in the market *(rank the control methods using proportional piling)*

- *Probe: i.e (As the animals are brought to the market what surveillance methods are in place in the markets?)*
- *do they quarantine, cull sick animals?*
- *Do they report sick or suspicious animals?*
- *What are their challenges with reporting of disease?*

12. What are the challenges you face in the prevention and control of diseases in small ruminants in the market? (*rank them using proportional piling)*

13. What are the recommendations for improving prevention and control of diseases in small animals in the market? (*rank them using proportional piling)*

*Thank you for participating in the study*

**FOCUS GROUP DISCUSSION GUIDE FOR TRANSHUMANCE GROUP**

**Study population:** Only 10 people per LGA will be purposefully selected) chosen after discussion with transhumance leader

**SECTION1: DEMOGRAPHICS/IDENTIFICATION**

Transhumance will identify themselves, age, experience, and market. Consent forms will be signed or consent will be given orally. GPS will be taken. Contact information will be taken if needed to contact for further information

Date: __________________        Code_____________   GPS____________

Name of interviewer___________ State___________     LGA: _______________ _ Village _ ________________            District___________ Name of the market: ________

**SECTION 2: ECONOMIC PURPOSES AND LOCATION OF HOTSPOTS**

1.   What are the purposes of small animals brought to the market? *(rank them using proportional piling*).

2.   List the fattening centres, slaughter houses/ abattoir, watering points, feeding points in the market/LGA.

**SECTION 3: ROUTES OF MIGRATION**

3. **When do you start migrating from this LGA?**

- Probe: Describe all your routes of migration?
- How long does it take you to get from this LGA to your resting point?
- How long do you usually spend at your resting point before coming back?
- When do you start coming back?
- What routes do you take as you start coming back?

**SECTION 4: LIVESTOCK MOVEMENT**

4.   Where do you buy most of your small ruminants from?

*(Fill the following on the flip chart)*

| Name of places | State | LGA | Village | Proportion |
| --- | --- | --- | --- | --- |
|  |  |  |  |  |
|  |  |  |  |  |

5.   Where do you go to sell your small animals *(use proportional piling)?*

*(Fill the following on the flip chart)*

| Name of places | State | LGA | Village | Proportion |
| --- | --- | --- | --- | --- |
|  |  |  |  |  |
|  |  |  |  |  |

6. When do you go to these markets to buy and sell small ruminants?

*(Fill the following on the flip chart)*

| *Name  of Market* | Jan | Feb | Mar | Apr | May | June | Jul | Aug | Sep | Oct | Nov | Dec |
| --- | --- | --- | --- | --- | --- | --- | --- | --- | --- | --- | --- | --- |
|  |  |  |  |  |  |  |  |  |  |  |  |  |
|  |  |  |  |  |  |  |  |  |  |  |  |  |

7.   Why do you choose these markets to buy and sell? *(Fill the following on the flip chart)*

| Market | Reason |
| --- | --- |
|  |  |
|  |  |

**SECTION 4: RETROSPECTIVE STUDIES**

*SEASONAL CALENDAR*

8. Seasonal calendar: How does the season affect the following factors?

Probe: In which months do you experience rainfall, dry season, more pasture, more presence of shared ponds, more animal births, more animal deaths?

- *Probe: When do you have more sheep and goat births and death proportion,*
- *When do you have the most pasture, rainfall period, animal density, animal movement?*
- *When do you have festivities? In what months do you have the most sales of sheep and goats ? In what months do you purchase the most sheep and goats?*
- *When do you normally observe PPR, when was the last outbreak of PPR in the markets, and in the LGA?*
- *When do you vaccinate your sheep and goat against PPR?*
- *When do transhumance come to you?*
- *When do you go to the transhumance?*

*(Fill the following on the flip chart)*

|  | Jan | Feb | Mar | Apr | May | June | Jul | Aug | Sep | Oct | Nov | Dec |
| --- | --- | --- | --- | --- | --- | --- | --- | --- | --- | --- | --- | --- |
| *Rainfall* |  |  |  |  |  |  |  |  |  |  |  |  |
| *Dry season* |  |  |  |  |  |  |  |  |  |  |  |  |
| *Pasture* |  |  |  |  |  |  |  |  |  |  |  |  |
| *Ponds* |  |  |  |  |  |  |  |  |  |  |  |  |
| *Biths* |  |  |  |  |  |  |  |  |  |  |  |  |
| *Deaths* |  |  |  |  |  |  |  |  |  |  |  |  |
| *Sale* |  |  |  |  |  |  |  |  |  |  |  |  |
| *Purchase* |  |  |  |  |  |  |  |  |  |  |  |  |
| *Festivities* |  |  |  |  |  |  |  |  |  |  |  |  |
| *PPR in market* |  |  |  |  |  |  |  |  |  |  |  |  |
| *PPR in LGA* |  |  |  |  |  |  |  |  |  |  |  |  |
| *Vaccination* |  |  |  |  |  |  |  |  |  |  |  |  |
| *Market prices are higher* |  |  |  |  |  |  |  |  |  |  |  |  |
| *Market prices are lower* |  |  |  |  |  |  |  |  |  |  |  |  |
| *When do the transhumance come to you* |  |  |  |  |  |  |  |  |  |  |  |  |
| *When do you go to the transhumance* |  |  |  |  |  |  |  |  |  |  |  |  |

**SECTION 5: DISEASE KNOWLEDGE, PREVENTION AND CONTROL MEASURES**

9. What are the priority diseases in small ruminants? (use a disease matrix table)

| DISEASE NAME | CLINICAL SIGN | SEASON | AGE | SEX | CAUSE | PREVENTION | TREATMENT |
| --- | --- | --- | --- | --- | --- | --- | --- |
|  |  |  |  |  |  |  |  |
|  |  |  |  |  |  |  |  |

*10.* Vaccination: what is their knowledge and use of vaccines, vaccine coverage for PPR and their recommendations for vaccine coverage.

11. From where do you get your vaccines from *(rank using proportional piling*)

12. What measures do you have to prevent and control the introduction of disease from sick animals in the market *(rank the control methods using proportional piling)*

- *Probe: i.e (As the animals are brought to the market what surveillance methods are in place in the markets?)*
- *Do they quarantine, cull sick animals?*
- *Do they report sick or suspicious animals?*
- *What are their challenges with reporting of disease?*

13. What are the challenges you face in the prevention and control of diseases in small ruminants in the market? (*rank them using proportional piling)*

14. What are the recommendations for improving prevention and control of diseases in small animals in the market? (*rank them using proportional piling)*

*Thank you for participating in the study*
